# Gel-Embedded Biochar and Hydroxyapatite Composite for the Improvement of Saline-Alkali Soil and Plant Growth Promotion

**DOI:** 10.3390/gels10040222

**Published:** 2024-03-25

**Authors:** Xin Hu, Weiqin Ma, Lhamo Pasang, Jiansheng Li, Haoming Chen

**Affiliations:** School of Environmental and Biological Engineering, Nanjing University of Science and Technology, Nanjing 210094, China; xinhu@njust.edu.cn (X.H.); mwq0403@njust.edu.cn (W.M.); bala0508@njust.edu.cn (L.P.)

**Keywords:** biochar, hydroxyapatite, gel material, saline–alkali soil, plant growth

## Abstract

Soil amendments play a crucial role in modern agriculture, as they effectively enhance the planting environment. This study innovatively proposes the use of gel as a crosslinking agent to embed biochar and hydroxyapatite (HAP), thereby preparing a novel soil amendment. Furthermore, this study investigates the soil improvement effects of this amendment as well as its influence on plant growth. This study employed a hydrothermal method to combine corn stalk (CB) or sludge (SB) biochar with HAP at different ratios (0–20%). Subsequently, sodium alginate gel (SA) was utilized to encapsulate the biochar and minerals, successfully forming a ternary composite gel material (corn stalk biochar/sludge biochar–sodium alginate gel-hydroxyapatite: CB/SB-SA-HAP). Finally, the practical effectiveness of this amendment was verified through potted soil experiments. The results indicate that the CB/SB-SA-HAP composite materials exhibited a micrometre-scale spherical structure with well-developed micropores and possess the functional groups of CB/SB, SA, and HAP, along with unique mineral properties. Through pot experiments, it was verified that the composite material effectively enhances multiple soil properties. After 21 days of cultivation, the soil pH values stabilized within the neutral range (pH = 7 ± 0.3) across all treatment groups. Except for the CB0 (CB:HAP = 1:0) and CB2.0 (CB:HAP = 1:2) treatments, the remaining treatments significantly reduced the soil EC values by 3.27% to 47.92%. All treatments significantly increased the contents of alkali-hydrolysable nitrogen (AHN) (34.89~57.91%), available phosphorus (AP) (35.93~56.55%), and available potassium (AK) (36.41~56.80%) in the soil. In comparison, although the SB treatment was more effective in regulating the pH and electrical conductivity (EC) of saline–alkali soil than the CB treatment, it was less effective in promoting plant growth in the short term. Through correlation analysis and redundancy analysis, a significant positive correlation was found between soil pH and ryegrass germination rate and plant height, particularly with the most pronounced impact on soil pH observed in the CB1.0 and SB0 (SB:HAP = 1:0) treatments. This study underscores the potential of CB/SB-SA-HAP composite materials in soil improvement and plant growth promotion, providing valuable insights for soil remediation, enhancement, and plant cultivation advancements in the agricultural sector.

## 1. Introduction

Improving the quality of agricultural soil is crucial for increasing crop yield, eliminating rural poverty, and reversing natural resource degradation. Phosphorus (P), as one of the elements essential for agricultural production, ranks second only to nitrogen in terms of the most frequently limiting mineral nutrients for crop growth [1]. Therefore, exploring soil improvement technologies that can enhance the utilization rate of P fertilizer, increase crop yield, and effectively reduce phosphorus loss in the soil has become a crucial research topic in the field of agriculture [2]. Soil amendments, which are materials added to enhance soil fertility and its physical attributes, have been garnering increasing attention and research focus owing to their effectiveness and sustainability. By contrast, practices like crop rotation (including fallowing and minimum or no-tillage), green manure application, and water management, while important, can often be more complex and costly to implement. 

Hydroxyapatite (HAP), as a common P-containing mineral, has a wide range of sources and is non-polluting, environmentally friendly, and biocompatible [3]. Its ability to release PO_4_^3−^ makes HAP a frequently used soil additive, serving as a slow-release fertilizer. This release of PO_4_^3−^ results in the formation of slowly released phosphate compounds, while the remaining unbound PO_4_^3−^ directly enriches the soil with available phosphorus, thereby promoting plant growth [4]. Meanwhile, the excessive or frequent application of HAP may also pose a potential risk of water eutrophication by releasing P [5]. Therefore, scientists have made various attempts to improve the utilization efficiency of HAP in practical applications and reduce the environmental pollution risk of HAP, such as microbial activation [6], co-precipitation and calcining with silicate [7], citric acid surface modification [8], etc. Among these attempts, the use of inexpensive green restoration materials in combination with HAP has proven to be an environmentally friendly and cost-effective approach, significantly enhancing HAP’s potential in improving the quality of agricultural soils.

Biochar, an effective soil amendment for improving soil quality, is a highly stable carbon-rich material formed by pyrolysis under oxygen-limited or anaerobic conditions [9,10]. Research has demonstrated that biochar can promote crop growth by improving soil water retention, cation exchange capacity, basal soil respiration, and microbial biomass [11,12,13]. The rich porosity of biochar can also adsorb and retain micro-nutrients that are prone to leaching in the soil, thereby enhancing soil fertility (N, P, K, etc.) [14]. Recent studies have also highlighted biochar’s exceptional ability as a carrier for mineral complexation, where its well-developed pore structure facilitates the dispersion of aggregated minerals. This, in turn, enhances the contact and decomposition of minerals with soil components [4,15]. In summary, the combined application of biochar and HAP appears to be a synergistic strategy for improving soil quality. 

The nutrient content of biochar and its capacity to retain these nutrients are often influenced by the type of feedstock used. For example, biochar derived from the pyrolysis of cow dung at temperatures ranging from 300–600 °C exhibits an available phosphorus content that is 2.1–3.94 times higher compared to biochar produced from corn stalks [16]. Corn biochar demonstrates a significantly higher available potassium content (7987–25,707 mg/kg) than pig manure biochar (196–996 mg/kg) [17]. In addition, considering the stability and slow release of soil amendments in the application process, embedding with biodegradable polymer organic materials happens to be an effective measure. Sodium alginate gel (SA) is a natural polymer with good biodegradability and compatibility, and it is an ideal materials for controlling the release rate of nutrients [18]. At the same time, SA has been widely used to deliver biologically active substances and capture pollutants or nutrients in the environment, including agricultural soils [19,20]. Therefore, the malleability, adsorption, and slow decomposition of SA contribute to the retention and gradual release of nutrients in the composite soil conditioner itself and in the environment. The combined use of biochar, HAP, and SA for agricultural soil remediation/improvement may be a novel attempt.

The aim of this study was to investigate the improvement mechanisms of soil physicochemical properties and ryegrass growth when combining SA, biochar (corn stalk biochar/sludge biochar: CB/SB), and HAP. Firstly, we successfully prepared CB/SB-SA-HAP composite materials (microspheres) with different ratios using hydrothermal and droplet plastic methods. Secondly, a 21-day pot experiment was conducted to verify the short-term effects of the composite materials on soil nutrient elements and plant growth conditions. The characteristics of the composite materials were analysed using scanning electron microscopy [21], attenuated total reflection infrared spectroscopy (ATR-IR), and X-ray diffraction (XRD) techniques. Soil AHN, AP, and AK were studied using alkaline hydrolysis diffusion, NaHCO_3_ leaching, and NH_4_OAc leaching methods, respectively. Finally, correlation analysis and redundancy analysis were used to further reveal the relationship between environmental factors (soil property indicators) and ryegrass growth, thereby exploring the effects of the composite material on soil physical and chemical properties and ryegrass growth. This study combined the advantages of biochar, SA, and HAP to develop a new multi-component soil conditioner, providing reference for the research and application of soil conditioners.

## 2. Results and Discussion

### 2.1. Characterisation of CB/SB-SA-HAP Composites

**Figure 1:**
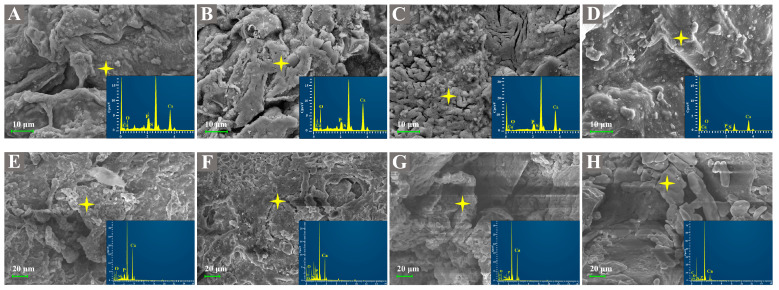
SEM diagram of biochar–gel-hydroxyapatite composite materials with eight proportions (**A**): CB0, (**B**): CB0.5, (**C**): CB1.0, (**D**): CB2.0; (**E**): SB0, (**F**): SB0.5, (**G**): SB1.0, (**H**): SB2.0; (CB = corn stalk biochar; SB = sludge biochar; yellow asterisk indicates the EDX spot scan position).

The SEM results of eight kinds of materials are shown in Figure 1. The surfaces of CB0 and SB0 were relatively smooth, with lines and no obvious furrows. The SA coating effect was significant (Figure 1A,E). After adding HAP, the surfaces of CB0.5–2.0 were rough, and there were obvious particles (Figure 1B–D). Among them, the surface of CB1.0 was the roughest, exhibiting a large number of agglomerates and an obvious pore structure. The SB treatment presented similar results to the CB treatment. In terms of morphology, the surface of SB0.5 had uniform particle coverage and no obvious agglomeration phenomenon (Figure 1F). SB1.0 had obvious furrows on the surface and larger particulate matter (Figure 1G). Compared with CB1.0 and SB1.0, fine particles increased, and gullies decreased on the surface of CB2.0. Similarly, SA in SB2.0 did not show a good effect of wrapping and covering materials, which might have been the result of massive aggregation on the material surface after increasing HAP. Excessive addition of HAP during the preparation process made it difficult to disperse the biochar. The agglomeration of fine particulate matter on the surface of biochar materials was the most obvious and clustered in one place (Figure 1H). In summary, with the increase in the proportion of the HAP composite, the surface of SB-SA-HAP composite microspheres showed a tendency to decrease in fine particulate matter, increase in grooves, and increase in agglomeration. SB’s own weaker specific surface area in comparison to CB might have been one of the main reasons for its lower ability to disperse minerals (SB BET = 13.62 m^2^/g, CB BET = 3.27 m^2^/g). The CB/SB-SA-HAP composite microspheres were rich in pores, and the biochar and HAP were successfully compounded and encapsulated within the SA.

The ATR-IR results showed that SA had obvious characteristic peaks at 3287 cm^−1^, 1595 cm^−1^, 1406 cm^−1^, and 1023 cm^−1^, which are representative of -OH, C=O, -COOH, and C-O [22]. There were multiple characteristic peaks in CB (1585, 879, 773 cm^−1^) and SB (1620, 820 cm^−1^), which belonged to the aromatic structures in biochar. These peaks were usually formed during the thermal decomposition of their raw materials. The characteristic peak at 1030 cm^−1^ belonged to the Si-O-Si asymmetric vibration in biochar. After SA and biochar were blended, the characteristic peaks of CB0/SB0 were basically consistent with those of SA and CB/SB, and the SA peaks had stronger signals, confirming that SA enclosed either CB or SB.

**Figure 2:**
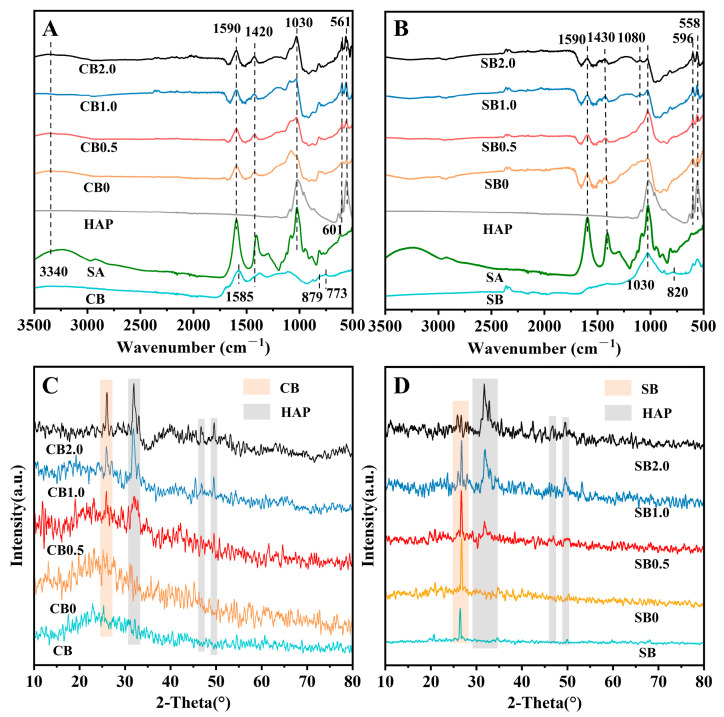
ATR-IR and XRD results of biochar–gel-hydroxyapatite composite materials (**A**,**B**): ATR-IR results of CB/SB–SA-HAP composites; (**C**,**D**): XRD results of CB/SB–SA-HAP composites; (CB = corn stalk biochar; SB = sludge biochar).

After adding HAP, CB/SB0.5–2.0 showed obvious phosphate ν4 P-O vibrations of HAP at 601 cm^−1^ (596 cm^−1^) and 561 cm^−1^ (558 cm^−1^) (Figure 2A,B). Additionally, with SA encapsulation, the characteristic peaks of biochar and HAP coexisted. With the increase in HAP, the peak intensities of biochar in the blended materials gradually decreased, which may be attributed to the dispersion of HAP over the surface of CB/SB. Compared with SB-SA-HAP, the peak intensities of all treatments in CB-SA-HAP were higher. The ATR-IR results confirmed that not only was HAP successfully loaded onto CB/SB but also SA successfully encapsulated CB/SB and HAP within the material.

The XRD results showed that compared with CB/SB0, CB/SB0.5–2.0 showed peaks of HAP at 2θ = 25.88°, 29.34°, 32.04°, 47.31°, and 49.36°. The peak signals of HAP were significantly enhanced as the compounding ratio of HAP increased (Figure 2C,D). The increase in the HAP compounding ratio did not affect the peak appearance of CB/SB. Therefore, SA was able to effectively encapsulate HAP and CB/SB without affecting the presence of their internal minerals. In addition, SA did not exhibit obvious mineral-like peaks. Its modification of the composites may be more focused on the optimization of the structure.

### 2.2. Soil Improvement Effect of CB/SB-SA-HAP Composites

#### 2.2.1. Soil pH and EC

**Figure 3:**
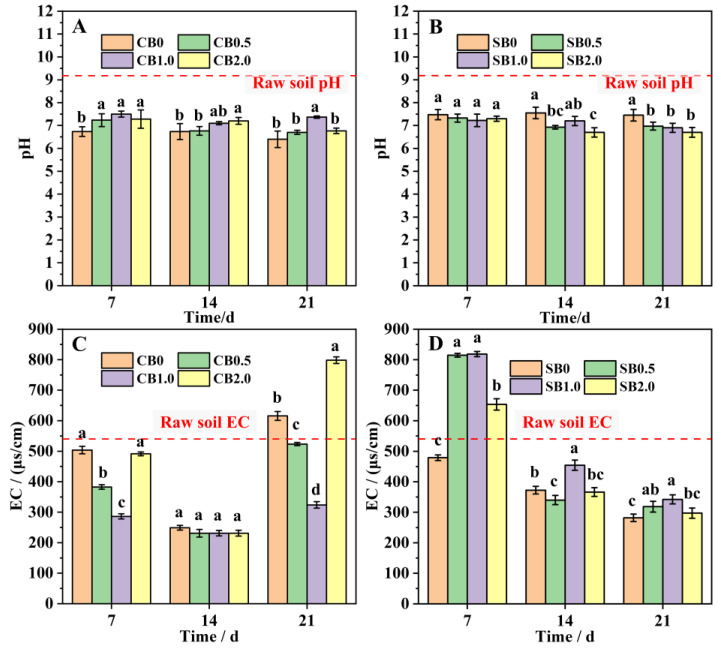
Soil pH and EC values after biochar–gel-hydroxyapatite composite materials improved soil. (**A**,**C**): Soil pH and EC values after CB–SA-HAP composites improved soil; (**B**,**D**): Soil pH and EC values after SB–SA-HAP composites improved soil; (CB = corn stalk biochar; SB = sludge biochar). All data points are the average of triplicate experiments. Error bars represents the standard deviation. Different Lowercase letters (a–c) indicate the statistically significant difference (*p* < 0.05) among different treatment groups at the same storage time.

The results of soil pH improvement showed that all eight treatments significantly reduced raw soil pH after 21 days, ultimately leading to a neutral soil condition (Figure 3A,B). In the CB-HAP-SA treatments, soil pH showed an initial increase followed by a decrease with the increase in HAP addition rate, with CB0 (pH = 6.40) having the lowest pH (Figure 3A). In the SB-SA-HAP treatments, pH showed a decreasing trend with increasing HAP addition rate, with SB2.0 (pH = 6.70) having the lowest pH (Figure 3B).

Except for CB0 and CB2.0, all the materials could effectively reduce the soil EC value after 21 days (Figure 3C,D). With the increase in time, the soil EC values of CB -SA-HAP treatments showed a tendency of decreasing and then increasing, and the soil EC values of all treatments reached the maximum at 21 days. Among them, soil EC under CB0 (615.67 μs/cm) and CB2.0 (798.33 μs/cm) treatments were higher than the original soil background value (EC = 541 μs/cm), while soil EC values of CB0.5 and CB1.0 were lower than the soil background EC by 3.27% and 40.11%, respectively. Soil EC values of SB-SA-HAP (0–2.0) treatments continued to decrease with time, and were finally (21 day) lower than the soil background EC by 36.74–47.92%. Therefore, CB/SB-SA-HAP complex microspheres have the ability to regulate soil alkalinity and salinity.

#### 2.2.2. Soil Alkali-Hydrolysable Nitrogen, Available Phosphorus, and Available Potassium

**Figure 4:**
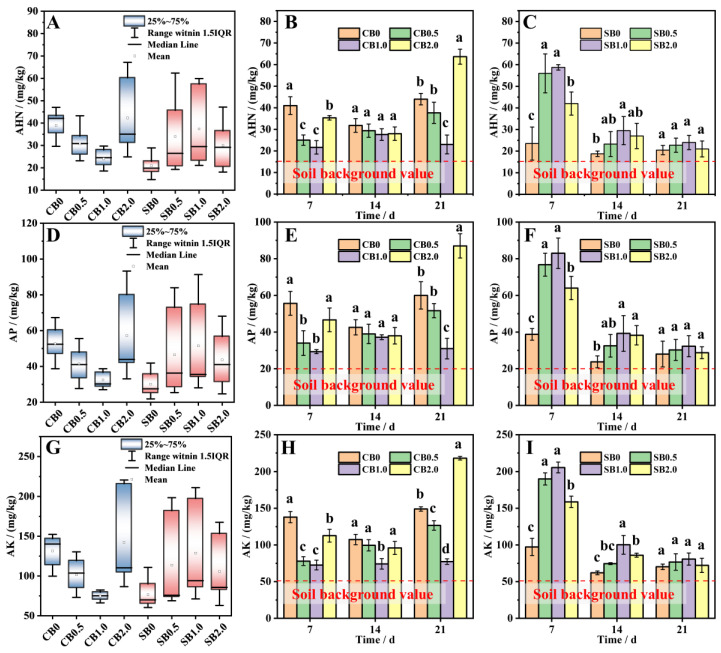
Alkali-hydrolysable nitrogen, available phosphorus, and available potassium contents after soil was modified by biochar–gel-hydroxyapatite composite materials. (**A**,**D**,**G**): Box-Plots of AHN, AP, and AK contents after treatment with CB/SB–SA-HAP composites; (**B**,**E**,**H**): AHN, AP, and AK contents after treatment with CB–SA-HAP composites; (**C**,**F**,**I**): AHN, AP, and AK contents after treatment with SB–SA-HAP composites; (CB = corn stalk biochar; SB = sludge biochar). All data points are the average of triplicate experiments. Error bars represents the standard deviation. Lowercase letters indicate variability among treat-ments at the same sampling time.

CB/SB-SA-HAP composite materials had a significant enhancing effect on the content of AHN, AP, and AK in the soil, and exhibited fluctuating changes with increasing experimental time (Figure 4). The fluctuation range of soil AHN, AP, and AK content over time in the CB-SA-HAP treatment group was smaller than that in the SB-SA-HAP treatment group. Among them, CB1.0 exhibited the best stability (Figure 4A,D,G). 

In the CB-SA-HAP treatment group, the content of soil AHN, AP, and AK showed a trend of first decreasing and then increasing with the increase in the proportion of HAP added. Throughout the experiment, the soil AHN, AP, and AK content of CB0.5 and CB1.0 were lower than those of CB0 and CB2.0. At 21 days, the soil AHN, AP, and AK contents in the CB2.0 treatment group were the highest, which were 1.45–2.77, 1.45–2.81, and 1.46–2.82 times those of the other three treatments, respectively (Figure 4B,E,H).

In the SB-SA-HAP treatment group, soil AHN, AP, and AK contents gradually decreased with time. Among them, SB0 had significantly lower AHN, AP, and AK than SB0.5 (AHN = 58.04%, AP = 49.51%, and AK = 48.82%), SB1.0 (AHN = 60.00%, AP = 53.31%, and AK = 52.68%), and SB2.0 (AHN = 44.05%, AP = 39.45%, and AK = 38.74%) at 7 days. At 21 days, the differences in soil AHN, AP, and AK contents among all the treatments did not differ significantly.

### 2.3. Effect of CB/SB-SA-HAP Composites on Ryegrass Growth

**Figure 5:**
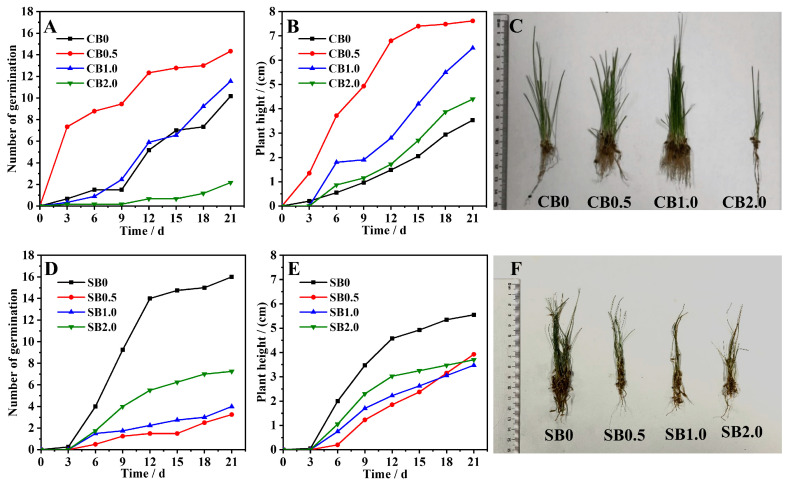
Germination rate, plant height, and plant morphology of biochar–gel-hydroxyapatite composite materials amended ryegrass at day 21. (**A**,**D**): Germination rate of ryegrass treated with CB-SA-HAP composites and SB-SA-HAP composites; (**B**,**E**): Plant height of ryegrass treated with CB-SA-HAP composites and SB-SA-HAP composites; (**C**,**F**): Pictures of ryegrass after 21 days of treatment with CB-SA-HAP composites and SB-SA-HAP composites; (CB = corn stalk biochar; SB = sludge biochar).

The CB/SB-SA-HAP composite materials significantly affected the growth of ryegrass (Figure 5). Ryegrass seeds began to germinate on the 3rd day (Figure 5A,D). On day 21, the germination rates of the CB-SA-HAP (0, 0.5, 1.0 and 2.0) treatment group were 25.42%, 35.83%, 28.89%, and 5.42%, respectively. The order of plant height was as follows: CB0.5 > CB1.0 > CB2.0 > CB0. Notably, the ryegrass plant height in the CB0.5 treatment group was 7.62 cm, which was 1.17–2.16 times that of CB0, CB1.0, and CB2.0 (Figure 5B). The germination rates of the SB-SA-HAP (0–2.0) treatment group were 40%, 8.13%, 10%, and 18.13%, respectively, with the highest ryegrass plant height observed in the SB0 treatment (Figure 5E). Additionally, on day 21, the root lengths of the ryegrass plants were in the following order: CB0 > CB2.0 > CB0.5 > SB0.5 > SB2.0 > CB1.0 > SB0 > SB1.0 (Figure 5C,F). Overall, CB0.5, CB1.0 and SB0 had the most significant promoting effects on ryegrass germination rate and plant height. The combination of biochar-SA-HAP has the potential to play a beneficial role in soil improvement and plant growth, suggesting that the amount of HAP added might not have been the dominant factor influencing the germination rate and plant height of the ryegrass seeds.

### 2.4. Mechanisms of the Effects of the CB/SB-SA-HAP Composites on Soil and Plants

**Figure 6:**
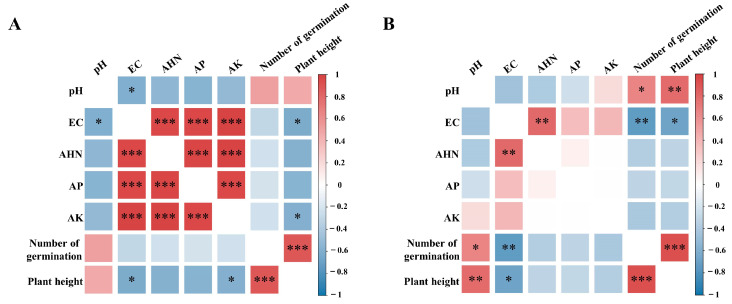
Heat map of the correlation between soil environmental factors and plant growth (**A**): CB-SA-HAP treatment group; (**B**): SB-SA-HAP treatment group; (21 days, CB = corn stalk biochar; SB = sludge biochar; * represents *p* < 0.05; ** represents *p* < 0.01; *** represents *p* < 0.001). “*” Stands for distinctiveness.

The correlation analysis between soil environmental factors and plant growth revealed that the trends in correlation between various soil environmental factors and plant growth were comparable for the two distinct types of biochar–SA–HAP compound materials (Figure 6). In the CB-SA-HAP and SB-SA-HAP treatment groups, soil pH exhibited positive correlations with ryegrass germination rate and plant height, with correlation coefficients of 0.51 and 0.47 for the CB-SA-HAP group and 0.66 and 0.79 for the SB-SA-HAP group. However, EC, AHN, AP, and AK exhibited negative correlations with both ryegrass germination rate and plant height. 

**Figure 7:**
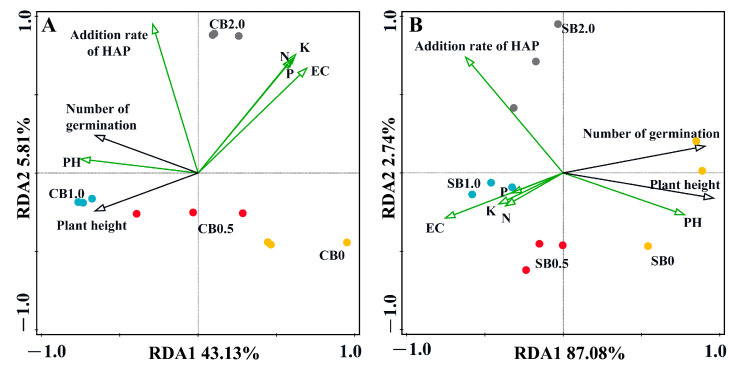
RDA analysis between soil environmental factors and plant growth for different treatments. (21 d, (**A**): CB-SA-HAP treatment group; (**B**): SB-SA-HAP treatment group; CB = corn stalk biochar; SB = sludge biochar).

The results of the RDA analysis revealed that the total explanatory power for both axes was 48.94% (43.13% ± 5.81%) under the CB-SA-HAP treatment and 48.94% (43.13% ± 5.81%) under the SB-SA-HAP treatment (Figure 7). The type of biochar and the amount of HAP added exerted distinct influences on environmental and biological factors. All treatments were distinctly positioned on the graph without any overlap. Within the CB and SB treatment groups, the arrows representing EC, AHN, AP, and AK all pointed in the same direction (indicating a positive correlation) and were positively correlated with CB2.0 (SB0.5 and SB1.0). Conversely, EC, AHN, AP, and AK demonstrated negative correlations with plant height and germination rate, owing to the depletion of soil nutrients by plant growth. It is worth noting that the positive effect of pH on ryegrass plant height/germination rate was the most significant, and CB1.0 and SB0 had the greatest effect on pH.

### 2.5. Effect of CB/SB-SA-HAP Composites on Plants by Regulating Soil pH and EC

Soil pH can affect the transformation of soil minerals and the forms of the existence of soil nutrients, ultimately influencing the soil’s nutrient supply capacity and the growth status of crops. Under both acidic and alkaline conditions, crop growth can be subject to stress [23]. CB/SB-SA-HAP treatments can effectively adjust soil pH, and the use of these specifically aimed to reduce alkalinity in this study (Figure 3A,B). Combined with a correlation heatmap analysis, it was discovered that a strong positive correlation existed between the change in pH and the growth of ryegrass. The extent of pH reduction may depend on the biochar type and HAP addition rate. Biochar itself also exerts a strong regulatory effect on soil pH. Both CB and SB are abundant in acidic functional groups, namely carboxyl and phenolic hydroxyl groups (Figure 2A,B). Furthermore, CB and SB indirectly contribute to lowering soil pH by enhancing the soil buffering capacity [24], facilitating the uptake of cations by plants (such as K^+^, Ca^2+^, and Mg^2+^) with concurrent H^+^ release [25], and boosting soil organic matter content [26]. Taking into account the initial pH values of CB and SB (Table 1), the incorporation of HAP led to a reduction in the pH of CB and SB composite materials by 35.5–36.0% and 1–5%, respectively. Meanwhile, the pH of both composites gradually decreased with the increase in HAP content, which may be due to the fact that the pH of HAP itself is lower than both the background soil pH (pH = 8.07) and that of biochar. Hence, we speculate that the adjustment effect of HAP on soil pH is more pronounced than that of biochar, which aligns with the findings from the RDA analysis (Figure 7).

The application of CB/SB-SA-HAP composite microspheres effectively reduced soil EC values within 21 days (Figure 3C,D). The change in EC may be primarily attributed to the properties of biochar. The capacity of biochar to mitigate the soluble salt content in soil and reduce soil EC has been demonstrated in numerous studies [27,28,29]. The porous structure and large specific surface area of biochar result in a high adsorption capacity, which can directly increase the adsorption of alkaline ions and thereby reduce soil EC. Specifically, the richer pore structure of CB compared to SB might have been one of the reasons for the lower EC value under CB-SA-HAP treatment in the early stage of the experiment (Day 7). Moreover, the soil EC values were also influenced by the feedback of crop growth. The germination rate and growth of ryegrass were significantly higher in the early stage (0–14 days) compared to the later stage (14–21 days), and this was accompanied by a more evident variation in soil EC during the early stage. During the early stages of the experiment, the observed reduction in EC values across all treatments can be attributed to a combination of cations absorbed by ryegrass during its growth and those adsorbed by the material. However, at the 21-day mark, ryegrass growth in the CB-SA-HAP treatment group started to show a decline, and the potential release of cations from the surface of the composite material could result in an increase in soil EC. In addition, although CB exhibits an abundant pore structure and a high specific surface area, its surface is relatively smooth compared to that of SB (as depicted in Figure 1). Therefore, there is a possibility that the salts, which were initially adsorbed and precipitated by CB-SA-HAP, would detach and be reintroduced into the soil. Throughout the later stages of cultivation (14–21 days), the electrical conductivity of the SB-SA-HAP treatment group remained consistently lower than the background soil electrical conductivity, showing a consistent decrease. This could be due to the effective adsorption and fixation of cations in the soil by the SB-SA-HAP composite. SB may possess a higher concentration of soluble anions that react with cations in the soil to form precipitates, indicating its potential superiority over CB in regulating soil salinity.

### 2.6. Effect of CB/SB-SA-HAP Composites on Plant Growth by Regulating Soil Nutrients

The incorporation of CB/SB-SA-HAP composite materials effectively elevated soil AHN, AP, and AK content (Figure 4). N, P, and K are pivotal elements involved in plant metabolism. The augmentation of soil AHN, AP, and AK in this study was primarily attributed to CB/SB. Biochar is inherently abundant in mineral nutrients such as N, P, K, Ca, and Mg [30]. Furthermore, the well-developed pore structure and substantial specific surface area of biochar facilitate its ability to enhance nutrient retention and optimize nutrient uptake and utilization efficiency when introduced into the soil [31].

As shown in Table 1, the addition of CB/SB was the main reason for the increase in AHN content. In addition, biochar has a strong adsorption capacity for NO^3−^ and NH^4+^, which is conducive to the retention of N in the soil during application [32]. During the pyrolysis and carbonization process, CB/SB retains a substantial amount of low-volatility P elements, predominantly in the form of soluble inorganic orthophosphates and polyphosphates. This retention directly contributes to augmenting soil P reserves when applied to the soil [33,34]. In this study, the increase in soil AP content under the combination of CB/SB and HAP can be primarily attributed to the abundant P content of HAP (Figure 4B,E). The availability of soil N and P is also influenced by pH. An excessively high pH (tested soil pH = 9.2 ± 0.3 in this study) can inhibit their availability [35]. The addition of CB/SB-SA-HAP composite materials aids in reducing soil pH. This, in turn, mitigates NH_3_ volatilization, enhances the solubility of inorganic P in the soil, and thus increases soil N and P content [24,36,37]. The notable elevation in soil AK primarily results from the preservation and subsequent conversion of K into highly soluble salts during the production of CB/SB [38]. CB/SB can also augment soil AK content through short-term interactions and reactions with the soil, encompassing processes such as dissolution and precipitation, adsorption and desorption, and redox reactions [26,39]. Notably, effective forms of N, P, and K can be directly absorbed by crop roots, further facilitating crop growth. This mechanism concurs with observations from this study, specifically the decrease in total soil AHN, AP, and AK levels corresponding with ryegrass growth (as depicted in Figure 5). For instance, in the CB1.0 (SB0) treatment, the levels of soil AHN, AP, and AK remained consistently lower over 21 d compared to those in the CB0, CB0.5, and CB2.0 (SB0.5–2.0) treatments. This confirms that ryegrass growth significantly depends on the absorption of nutrients from the soil. Both correlation and redundancy analyses further substantiate the robust correlation between AHN, AP, AK, and ryegrass growth.

In summary, CB/SB-SA-HAP composites can effectively enhance saline–alkali soil physical and chemical properties (EC, pH, AHN, AP, AK) while promoting ryegrass growth. Regardless of whether it is the CB treatment group or the SB treatment group, the influence of pH on plant growth surpasses that of nutrient elements. While pH is a critical factor, the availability of key nutrients also plays a significant role in plant growth promotion. Correlation and redundancy analysis further affirm that the CB-SA-HAP material primarily boosts plant growth through the provision of N and K, whereas the SB-SA-HAP material primarily facilitates growth through the supply of N, P, and K. Therefore, the rational application of conditioner materials, such as CB/SB, is pivotal for augmenting soil fertility and fostering crop growth.

## 3. Conclusions

The results of this study indicate that CB/SB-SA-HAP composite material can effectively decrease soil pH and EC while enhancing soil AHN, AP, and AK content. Both CB/SB-SA-HAP composite materials effectively promoted the growth of ryegrass, thereby facilitating the absorption and utilization of nutrients. The SB-SA-HAP composite materials demonstrated superior regulation of soil pH and EC compared to the CB-SA-HAP composite material. In the short term, the CB-SA-HAP composite materials were more beneficial for plant growth in terms of soil nutrient provision than SB-SA-HAP. The decreasing effect of plant growth with increasing HAP in the SB treatment group may be attributed to insufficient nutrient release. Therefore, the rational use of CB-SA-HAP and SB-SA-HAP composite materials holds potential for soil improvement/fertilization.

## 4. Materials and Methods

### 4.1. Experimental Materials and Soil

The biochar used in this experiment was made from corn stalks and sludge, which were obtained from farmlands in Huaian, Jiangsu Province and a sewage treatment plant in Yongkang, Zhejiang Province, respectively. The corn stalks and sludge were first washed with pure water and anhydrous ethanol 2 times, then placed in a 70 °C oven for 24 h to dry. After drying, they were ground and sieved (<100 mesh). The ground raw materials were then carbonized in a muffle furnace at 450 °C (for corn stalks) and 600 °C (for sludge) for 40 min (with a heating rate of 5 °C/min). After natural cooling, the carbonized materials were washed with pure water and anhydrous ethanol 2 times and then dried to constant weight to obtain the biochar (CB/SB). The production details can be referred to our previous studies [10]. The soil used in this experiment was collected from saline–alkali sandy soil in Shandong Province (118.78112° E, 37.83379° N), which was naturally air-dried, ground, and sieved (≤70 mm) for use. The ryegrass seeds were obtained from the Jiangsu Academy of Agricultural Sciences. The basic physical and chemical properties of the materials and soil are shown in Table 1.

### 4.2. Preparation of Biochar and Biochar-SA-HAP Composites

**Figure 8:**
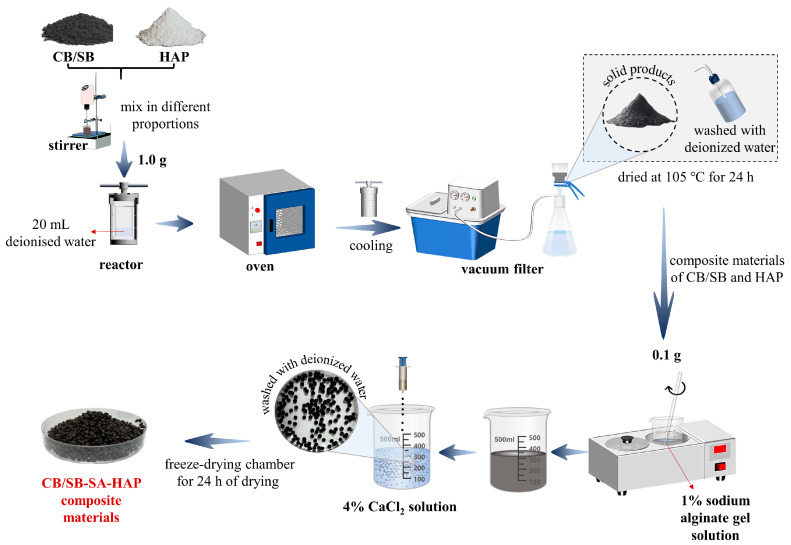
Schematic diagram of the preparation process of biochar–gel-hydroxyapatite composite materials.

The CB/SB was mechanically mixed with HAP powder in different proportions (CB/SB:HAP = 1:0/0.5/1/2) in a glass beaker using a stirrer (100 rpm, 15 min). Then, 1.0 g of the mixture was added to a high-pressure reactor (capacity: 100 mL) containing 20 mL of deionized water. The high-pressure reactor was placed in an oven and heated to 180 °C at a rate of 5 °C per minute. The temperature was maintained for 20 min to allow for a better loading of HAP onto the CB/SB. After cooling to room temperature, the solid products were separated using a vacuum filter. It was washed with deionized water and then dried at 105 °C for 24 h to obtain the composite materials of CB/SB and HAP. Taking 0.1 g of the composite material, it was added to a solution of 1% SA (0.1 g of SA dissolved in 10 mL of deionized water and stirred in a 70 °C water bath for 20 min). The mixture was ultrasonically treated for 15 min (20 °C) to obtain the mixed solution. The mixed solution was taken with a syringe (2 mL) and added dropwise to a 4% CaCl_2_ solution. After solidification, the materials were repeatedly washed with deionized water to remove Ca^2+^ from the materials and then placed in a freeze-drying chamber for 24 h of drying. Finally, different ratios of CB/SB-SA-HAP composite materials (microspheres) were obtained (Figure 8). According to the proportion of HAP added, they were denoted as CB0, CB0.5, CB1.0, CB2.0, SB0, SB0.5, SB1.0, and SB2.0, respectively.

### 4.3. Potting Experiments

After mixing 4.0 g of biochar-based composite materials with 196.0 g of soil, they were evenly distributed in culture pots (each pot was lined with cheesecloth at the bottom to prevent soil loss). Each pot was irrigated with deionized water until saturation, and then the prepared pots were placed in an artificial climate incubator for 1 day of equilibration (8 h of light, 20,000 LX light intensity, and 25 °C temperature). The following day, 40 ryegrass seeds were evenly sown on the surface of each pot, and the surface was sprayed with deionized water. The pot experiment lasted for 21 days. There were 8 treatments in total, with 3 replicates. During the experiment, lost water was replenished with deionized water every 3 days to maintain the soil moisture at 60% of the maximum water-holding capacity. All treated pots were randomly arranged, and the positions of the pots were randomly rotated every 3 days to ensure consistent environmental conditions for the planting treatments.

### 4.4. Testing and Analysis

#### 4.4.1. Material Characterisation Analysis

The morphology and elemental content of the samples were analysed by scanning electron microscopy (SEM, Hitachi Regulus 8100, Tokyo, Japan) and energy-dispersive X-ray spectroscopy (EDX, INCA 300 Oxford, UK). The functional group types of the materials were analysed by attenuated total reflectance infrared spectroscopy (ATR-IR, iD7 ATR, Thermo Fisher Scientific Inc., Madison, WI, USA). Mineral types in the materials were determined and analysed with an X-ray diffractometer (XRD, D8-Advance, Bruker, Ettlingen, Germany) and JADE 6.0. ATR-IR, XRD, and other graphs were plotted using Origin 8.5.

#### 4.4.2. Analysis of Basic Soil Physicochemical Properties and Plant Growth Conditions

The physicochemical properties of soil, such as AHN, AP, AK, pH, and electrical conductivity (EC), were analysed after 21 d of potting culture. Soil AHN was determined by the alkaline diffusion method [40]. The AP (NaHCO_3_) and AK (NH_4_OAc) of the soil were determined by leaching method. The solid–liquid mixture was centrifuged and filtered (10 mL, 5000 rpm/min, 3 min, 0.22 μm membrane) and then measured by an Inductively Coupled Plasma Optical Emission Spectrometer (ICP-OES, icap7000, ThermoFisher Scientific Inc. iCAP PRO, Waltham, MA, USA). Soil pH was determined using a pH meter (water/soil ratio of 2.5:1, PHBJ-260, Leimagnet, Shanghai, China). Soil EC was determined using a conductivity meter (water/soil ratio of 1:1, DDBJ-350, Ray Magnetics, Huzhou, China). Additionally, the plant height and germination rate were measured during the cultivation process.

#### 4.4.3. Statistics and Analysis

The correlation between the material properties of biochar, the physicochemical properties of the amended soil, and plant characteristics was analysed using principal component analysis (Canoco 5.0). Differences in nutrient content and plant growth among treatments were analysed via one-way ANOVA (Duncan’s test, SPSS 22.0, *p* < 0.05).

## Figures and Tables

**Table 1:** Basic physical and chemical properties of raw soil and CB/SB and HAP.

Basic Nature	pH	Alkali-Hydrolyzable Nitrogene (AHN) (mg/kg)	Available Phosphorus (AP) (mg/kg)	Available Potassium (AK) (mg/kg)	C (%)	N (%)	S (%)
Raw soil	9.2 ± 0.3	15.2 ± 2.0	20.6 ± 1.8	51.5 ± 3.6	--	--	--
CB	10.02 ± 0.1	14.7 ± 1.3	242.082 ± 16.1	811.768 ± 26.5	49.03	0.96	0.06
SB	6.75 ± 0.1	465.5 ± 11.6	753.71 ± 18.3	179.85 ± 9.3	14.08	1.77	0.35
HAP	8.07 ± 0.2	ND	139.13 ± 0.3	109.15 ± 10.3	--	--	--

Note: “ND” stands for not detected, “--” stands for not measured.

## Data Availability

The original contributions presented in the study are included in the article, further inquiries can be directed to the corresponding authors.
